# Self-Compassion Buffers the Adverse Mental Health Impacts of COVID-19-Related Threats: Results From a Cross-Sectional Survey at the First Peak of Hong Kong's Outbreak

**DOI:** 10.3389/fpsyt.2020.585270

**Published:** 2020-11-05

**Authors:** Bobo Hi-Po Lau, Cecilia Lai-Wan Chan, Siu-Man Ng

**Affiliations:** ^1^Department of Counselling and Psychology, Hong Kong Shue Yan University, Hong Kong, China; ^2^Department of Social Work and Social Administration, University of Hong Kong, Hong Kong, China

**Keywords:** self-compassion, mental health, perceived benefit, COVID-19, Hong Kong, self-coldness

## Abstract

COVID-19 has brought tremendous and abrupt threats to various aspects of our daily lives, from school and work to interpersonal relationships. Self-compassion is put forth as a salutogenic perspective on oneself that buffers the adverse mental health impacts of these threats. During the peak of a local outbreak in Hong Kong in Spring 2020, 761 participants completed questionnaires on self-compassion, perceived threats, as well as perceived benefits and psychological distress. Controlling for demographic variables, negative indicators of self-compassion (aka self-coldness) was found to intensify the impacts of threats on psychological distress. The positive indicators of self-compassion also moderated the link between threats and perceived benefits, such that perceived benefits tend to be less related to threats in participants with higher self-compassion. Our findings highlight the impacts of both positive and negative indicators of self-compassion on the adjustment to such unprecedented challenges, and point to the possibility of enhancing people's resilience through fostering self-compassion and alleviating self-coldness.

## Introduction

The Coronavirus Disease-2019 (COVID-19) has imposed unprecedented changes to our everyday lives. In countries where cities were locked down, citizens were forced into furlough or work-/school-at-home arrangements with wide-spread suspension of services and businesses, entailing pervasive loneliness, and sense of insecurity (1, 2). The perfect storm from coupling psychological tension with 24/7 interactions with one's family in an enclosed space breeds conflicts or even domestic violence (3).

This study was conducted during the peak of the Spring 2020 outbreak in Hong Kong, when public health orders banning public gatherings, restricting catering capacity of restaurants to half and mandating closure of high-risk businesses were enforced for the first time after reports of infection clusters in March. Conceivably, these measures have drastically changed the citizens' daily routines and resulted in enormous challenges to the local businesses and the civil society, especially after months of conflicts during the Anti-extradition law amendment bill (Anti-ELAB) movement (4). In fact, Hong Kong experienced the worst drop in year-to-year GDP (8.9%) during Spring 2020 where the worst wave of local outbreak occurred, whilst the unemployment rate has reached a 15-years high (5.9%) (5). The ban on mass gatherings and apprehension over physical socializing has also hindered connections among stakeholders of the civil society, adversely impacting the community support networks. In such unprecedentedly challenging time, this study was conducted to explore how self-compassion, defined as a warm, kind and non-judgmental attitude to oneself during setbacks, modulates individuals' adjustment to the pandemic-related threats (6).

Neff proposed that self-compassion entails (i) extending kindness and understanding to oneself rather than treating oneself with harshness and criticism (self-kindness vs. self-judgment), (ii) seeing one's suffering as a part of the shared human experience rather than an isolated experience (common humanity vs. isolation), and (iii) a balanced perspective of one's suffering rather than overly attached to it (mindfulness vs. over-identification) (6). Accordingly, meta-analyses have reported robust negative associations of self-compassion with psychopathology and positive associations with well-being (7–9).

Self-compassion may modulate how people confront threats by encouraging adaptive coping responses. Allen and Leary summarized the associations between self-compassion and coping styles (10). Self-compassion tends to foster positive reappraisal and proactive coping and reduce avoidant behaviors. Evidence regarding self-compassion's salutary effects on problem-solving, support seeking and distraction was however mixed. Allen and Leary postulated that these associations could be qualified by perceived control, such that people with higher self-compassion exhibit higher proactivity (vs. passivity) when perceived control is higher (vs. lower) (10). Another line of research suggests that self-compassion induces favorable emotional regulation (e.g., emotion clarity, impulse control, acceptance of emotional response) which in turn engenders mental health benefits (11, 12). From a self-regulation perspective, self-compassion may facilitate healthy attainment of goals by facilitating proactivity, enabling one to take responsibility to both success and failure, evaluating the situation with equanimity, disengaging from relentless pursuits and countering the toxic effects of guilt and embarrassment (13–15). Accordingly, self-compassion moderated the impacts of stressors on well-being and adjustment in various samples, including women with breast cancer, college students, women with restricting eating tendencies, and even in a laboratory-induced stressful setting (16–19).

Self-compassion is often assessed with the full- or short-version of Self-Compassion Scale [SCS; (20, 21)]. Both versions assume a higher-order single factor structure and a six-factor structure encompassing three positive (self-kindness, mindfulness, common humanity) and three negative (self-judgment, over-identification, and isolation) factors. Thus, responses on the negative indicators are often reversed to attain an overall scale score of self-compassion. However, the construct validity of these scales is contentions, as many studies failed to replicate the six-factor model, but instead, yielded a bifactor structure with distinct but related positive (self-compassion/self-reassuring) and negative factors (self-coldness/self-criticism) (22–24).

Moreover, the positive and the negative factors appear to be asymmetrically related to psychopathology and well-being. Muris and Petrocchi found that while the positive and the negative indicators are related to psychopathology in expected directions, comparisons over the strengths of the relationships revealed the negative indicators as significantly stronger predictors than the positive ones (9). Such an observation indicates the possibility of an inflated association between self-compassion and psychopathology when the overall scale score, with the oppositely-phrased items reversed, has been used. In fact, the tendencies to be reassuring vs. critical to oneself rely on distinct biological impetuses. Longe et al. found that self-reported measures of self-criticism were associated with areas for error-processing and behavioral inhibition, including the dorsolateral prefrontal cortex, and those of self-reassurance with areas of empathy, including the ventrolateral prefrontal cortex (25). Accordingly, Brenner et al. proposed a theoretical model of self-relating based on Gilbert's theories of social mentalities (26–28). While self-compassion, which stems from a safeness system rooted in the parasympathetic nervous system, infers a non-judgmental, caring lens to own sufferings and therefore encourages positive connections to oneself and others; self-coldness, which stems from a threat-defense system rooted in the sympathetic nervous system, indicates a tendency to be critical, judgmental and overly attached to one's suffering, and exhibit vigilance or avoidance in behaviors toward others.

A bifactor model that distinguishes self-compassion from self-coldness may better fit how Asians affectively evaluate things in general. For instance, a dialectical thinking style, which has roots in Asian philosophies and religions (e.g., Confucianism and Buddhism), facilitates tolerance and flexible integration of affectively opposite judgments and coping strategies (29, 30). The Chinese circumplex model of affect also postulates the positive and the negative affect as independent but associated constructs, rather than two poles on the same line (31). Hence, we reckon that in the ethnic context of Hong Kong, it may make more sense to assume individuals can exhibit both self-compassion and self-coldness, although likely to different degrees and on different aspects of even the same event. For instance, one can be compassionate about one's worsening job prospect due to the financial meltdown, but still be self-critical about not being industrious enough to follow up with every client.

In this study, we tested the moderation effects of self-compassion and self-coldness simultaneously on the impacts of pandemic-related perceived threats on well-being. Also, as self-compassion and self-coldness may be differentially associated with well-being and psychopathology, we tested on outcomes indicating both negative and positive adaptations (26). The negative impacts were indicated by psychological distress that encompasses symptoms of anxiety and depression. Perceived benefits experienced in the pandemic were used to indicate positive adaptation to the challenging situation (32, 33). We expected self-compassion to buffer the positive relationship between perceived threats and psychological distress, as well as the negative relationship with perceived benefits. In other words, individuals with higher self-compassion should experience less emotional harm from threats and that their perceived benefits will be less hampered by threats. Brenner et al. put forth self-coldness as a separate vulnerability factor (26). We therefore anticipated self-coldness to intensify the positive relationship between perceived threats with psychological distress and the negative relationship with perceived benefits. That is, individuals with higher self-coldness should be more susceptible to the emotional harm from perceived threats and that their perceived threats should hamper perceived benefits more.

## Methods

### Design

This is a part of a longitudinal study on how people of Hong Kong adjust to the COVID-19 pandemic. The current analysis utilized only the cross-sectional data collected between mid-March to early-April 2020, right after the World Health Organization declaring COVID-19 a pandemic (34). The study was approved by the Human Research Ethics Committee of the University of Hong Kong (EA2003003).

### Participants

Adults aged 18 or above residing in Hong Kong were eligible for the study. Participants who could not read traditional Chinese or had no access to the internet were excluded. Conducted as a swift response to the situation, participants were recruited through snowballing by social media and email lists. Participants were reimbursed HKD$50 in cash or supermarket coupons for participating in the current survey.

### Instruments

#### Perceived Threats

Participants were asked to rate the extent to which the pandemic has rendered threats to their (i) work/academic life, (ii) personal finance, (iii) family relationships, and (iv) social life on a 10-point scale running from 1 (not at all) to 10 (extremely). The four self-constructed items were averaged to form an overall perceived threats scale, with good reliability (Cronbach α = 0.79).

#### Self-Compassion and Self-Coldness

The 12-item Self-Compassion Scale Short Form [SCS-SF; (21)] were used. These twelve short-form items were drawn from the published translation of the Chinese Self-Compassion Scale (35). Participants answered on a 5-point scale running from 1 (almost never) to 5 (almost always). The positive subscale (*self-compassion*) included the six items on self-kindness, mindfulness and common humanity, whereas the negative subscale (*self-coldness*) encompassed the six items on self-judgment, over-identification and isolation. The subscale scores were obtained by taking the average across responses on the items. The reliability of the two subscales were adequate with Cronbach alphas of 0.83 (positive) and 0.81 (negative), respectively.

#### Psychological Distress

Psychological distress over the past 2 weeks was measured by the Patient Health Questionnaire-4 [PHQ-4; (36)]. The first two items indicated anxiety levels and were taken from the Generalized Anxiety Disorder-7 [GAD-7; (37)]; while the last two items were from the Patient Health Questionnaire-2 (PHQ-2) that has been used for screening depression (38). These items were drawn from the published translation of the Chinese GAD-7 and PHQ-2 (39, 40). Participants answered on a four-point scale running from 0 (not at all) to 3 (nearly everyday). A summed response exceeding 5 indicates moderate to severe psychological distress. The reliability of the scale was good (Cronbach α = 0.87).

#### Perceived Benefits

Eleven self-constructed items were employed to indicate perceived benefits experienced by our participants during the pandemic (34). Example items include “The pandemic afforded me more time for rest and relaxation,” “I learned a new skill/knowledge from the pandemic,” and “I gained greater trust in the power of the citizens.” Participants answered on a seven-point scale running from 1 (strongly disagree) to 7 (strongly agree). The scale score was derived by taking the average across the responses on the items. The scale exhibited good reliability (Cronbach α = 0.86).

Demographic information, such as gender, age, education background, marital status, income, religion as well as health and pandemic exposure related data, such as presence of a chronic health condition, co-residence with an individual vulnerable to a severe course of illness in case of infection (e.g., children, elderly, individuals with chronic illness, pregnant women, etc.), level of risk at occupational setting, SAR-CoV-2 test results (if any) and medical quarantine experience were also collected from the online survey. The online survey was conducted in traditional Chinese.

### Statistical Analyses

Descriptive statistics were used to explore the levels of perceived threats, self-compassion, self-coldness, perceived benefits and psychological distress of the participants, while the intercorrelations among the key variables were scrutinized by Pearson's correlations. The moderating role of self-compassion and self-coldness were tested with SPSS PROCESS macro (version 3). Assuming a moderate effect size (*f*^2^ = 0.15), alpha of 0.05, power of 95% and 13 predictors (control variables, predictor variables and two interaction terms), a minimum of 189 participants were needed based on the calculation by G^*^Power (version 3.1.9.2). The current sample size exceeded what is minimally required for testing the model. The directions of the moderation effects were indicated by the effects of the focal predictor (i.e., perceived threats) on the outcome (i.e., psychological distress and perceived benefits) at 16th, 50th, and 84th percentile of the moderators (i.e., self-compassion and self-coldness). All analyses were conducted with SPSS (version 25.0).

## Results

### Sample Characteristics

Among the 761 participants (Table 1), 67.7% were female with age ranging from 18 to 79 [Mean (SD) = 40.31 (14.02)]. 62.4% of the sample received university education or above and 49.9% were married. 47.0% were affiliated to a religion. The median monthly family income was HKD$40,000–49,999, which was higher than the population median (HKD$27,000). About one-third of the participants (35.7%) reported they were working in a high-risk occupation (e.g., healthcare, retail, catering, and beverage, jobs that require frequent travel abroad). A quarter (24.6%) declared having at least one chronic physical or psychological illness, while about half of the sample (50.6%) were living with individuals vulnerable to a severe course of illness in case of COVID-19 infection. There were seven cases (0.9%) of positive test results of SAR-CoV-2 and 11 (1.4%) cases subjected to medical quarantine.

**Table 1:** Sample characteristics (*N* = 761).

	**N/*M***	**Valid %/*SD***
**Gender**		
Female	515	67.7
Male	246	32.3
**Age**	*40.31*	*14.02*
**Education backgrounds**		
Primary or less	6	0.8
Secondary	157	20.6
Higher diploma/Associate degree	123	16.2
Undergraduate	262	34.4
Post-graduate or above	213	28.0
**Marital status**		
Single, divorced, separated, bereaved	381	50.1
Married	380	49.9
**Income (in Hong Kong Dollar)**		
<10,000	57	7.5
10,000–19,999	91	12
20,000–29,999	107	14.1
30,000–39,999	89	11.7
40,000–49,999	86	11.3
50,000–69,999	132	17.4
70,000–89,999	85	11.2
90,000 or more	114	15
**Religion**		
Yes	358	47
No	403	53
**Own chronic health condition**		
Yes	187	24.6
No	574	75.4
**Live with a vulnerable person**		
Yes	385	50.6
No	376	49.4
**In a high-risk occupation**		
Yes	272	35.7
No	489	64.3

*Italics refers to M/SD = (means/standard deviation)*.

Both perceived threats and benefits from COVID-19 were moderate [Means (SDs) = 4.89 (2.10), 4.65 (0.99), respectively]. The mean of PHQ-4 was 3.29 (*SD* = 2.91). The percentages of participants showing none (score 0–2), mild (score 3–5), moderate (score 6–8), and severe (score 9–12) psychological distress were 46.3, 35.9, 11.4, 6.4%, respectively. In other words, 17.8% of the sample scored above the cut-off for moderate to severe psychological distress.

Table 2 shows the intercorrelations among the key variables. The negative association between self-compassion and self-coldness was moderate in magnitude. Perceived threats were positively related to psychological distress and self-coldness, but negatively associated with self-compassion. Self-compassion and self-coldness were negatively and positively related to psychological distress, respectively. Of note, perceived benefits were unrelated to perceived threats and psychological distress, but were positively and negatively correlated with self-compassion and self-coldness.

**Table 2:** Intercorrelations among the key variables (*N* = 761).

	**Mean (SD)**	**1**	**2**	**3**	**4**	**5**
1. Self-compassion	4.89 (2.10)	1.00	–	–	–	–
2. Self-coldness	3.59 (0.68)	−0.28^***^	1.00	–	–	–
3. Perceived threats	3.19 (0.73)	−0.16^***^	0.28^***^	1.00	–	–
4. Psychological distress	3.29 (2.91)	−0.29^***^	0.40^***^	0.41^***^	1.00	–
5. Perceived benefits	4.65 (0.99)	0.32^***^	−0.11^**^	0.06	−0.06	1.00

*SD, standard deviation*.

* p < 0.05;

** p < 0.01;

*** *p < 0.001*.

### Moderating Roles of Self-Compassion and Self-Coldness

**Figure 1:**
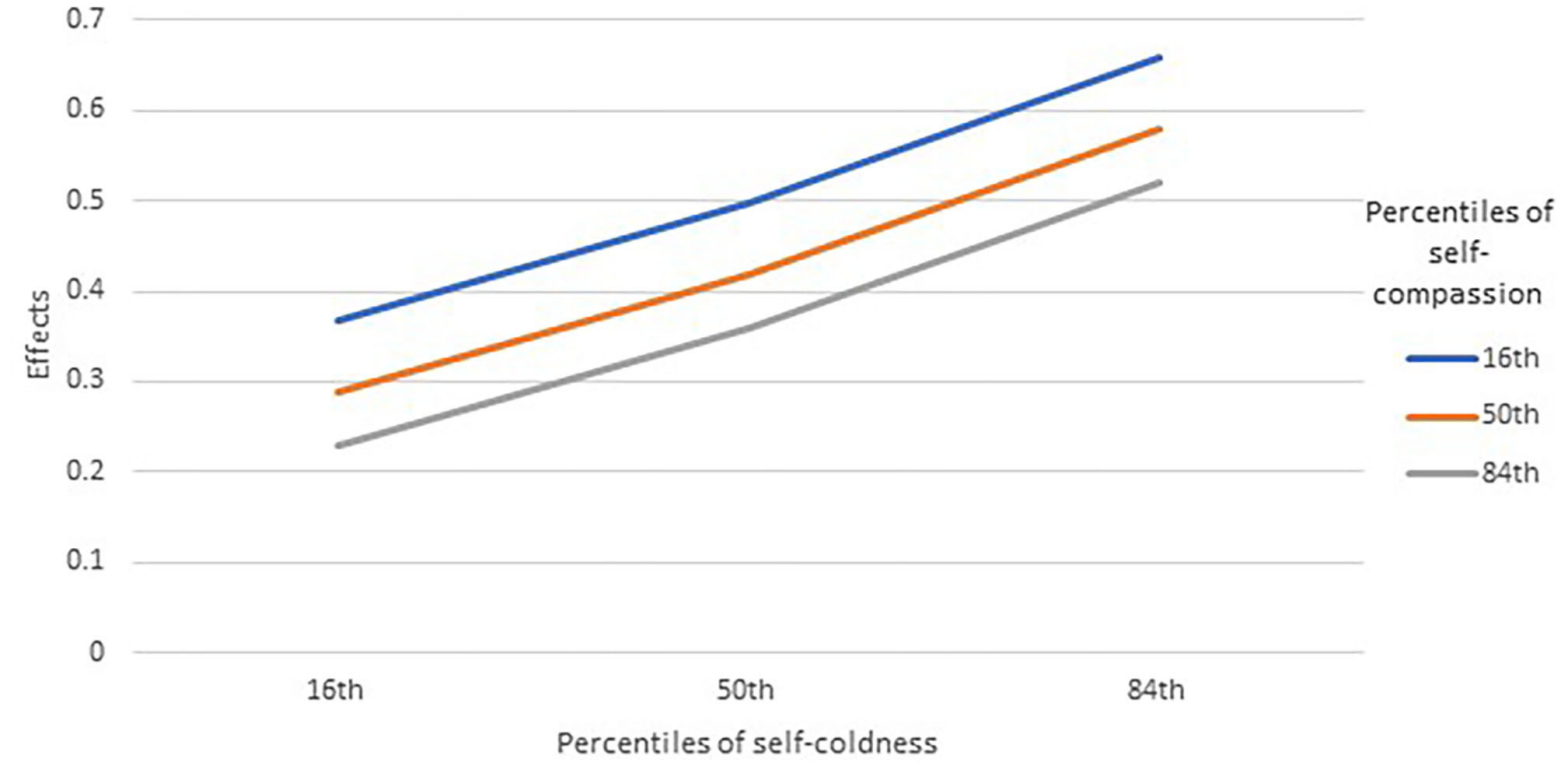
Moderation roles of self-compassion and self-coldness on the effects between perceived threats and psychological distress (*N* = 761). Effects were unstandardized coefficients of the conditional effects of perceived threats on psychological distress.

Table 3 provides the results of the moderation models. For psychological distress, the relationship with perceived threats was significantly moderated by both self-coldness (*p* = 0.0009) and self-compassion (*p* = 0.0439). Inspecting the effects of perceived threats on psychological distress at the 16th, 50th, and 84th percentiles of the moderators (Figure 1), self-coldness appeared to strengthen the positive association between perceived threats and psychological distress, while higher self-compassion was related to weaker positive association. The main effects of perceived threats, self-compassion, and self-coldness were non-significant.

**Table 3:** Results of moderation models (*N* = 761).

	**Psychological distress**	**Perceived benefits**
	**B (SE)**	**B (SE)**
Female (vs. male)	0.36 (0.19)	0.20 (0.07)^**^
Age	−0.00 (0.01)	0.00 (0.00)
University educated (vs. no)	0.08 (0.21)	−0.01 (0.08)
Income	−0.00 (0.03)	−0.01 (0.01)
Presence of own chronic health problems (vs. no)	0.25 (0.22)	0.02 (0.08)
Co-living with a vulnerable individual (vs. no)	0.25 (0.18)	0.02 (0.07)
Religious affiliation (vs. no)	−0.24 (0.18)	0.04 (0.07)
In a high-risk occupation (vs. no)	0.05 (0.19)	−0.05 (0.07)
Perceived threats	0.24 (0.29)	0.34 (0.11)^**^
Self-coldness	0.13 (0.30)	0.09 (0.11)
Self-compassion	−0.15 (0.32)	0.73 (0.12)^***^
Self-coldness × perceived threats	0.19 (0.06)^***^	−0.03 (0.02)
Self-compassion × perceived threats	−0.12 (0.06)^*^	−0.05 (0.02)^*^
Model summary: Δ*r*^2^	0.2968^***^	0.1412^***^

*B, unstandardized coefficient; SE, standard error*.

* p < 0.05;

** p < 0.01;

*** *p < 0.001*.

**Figure 2:**
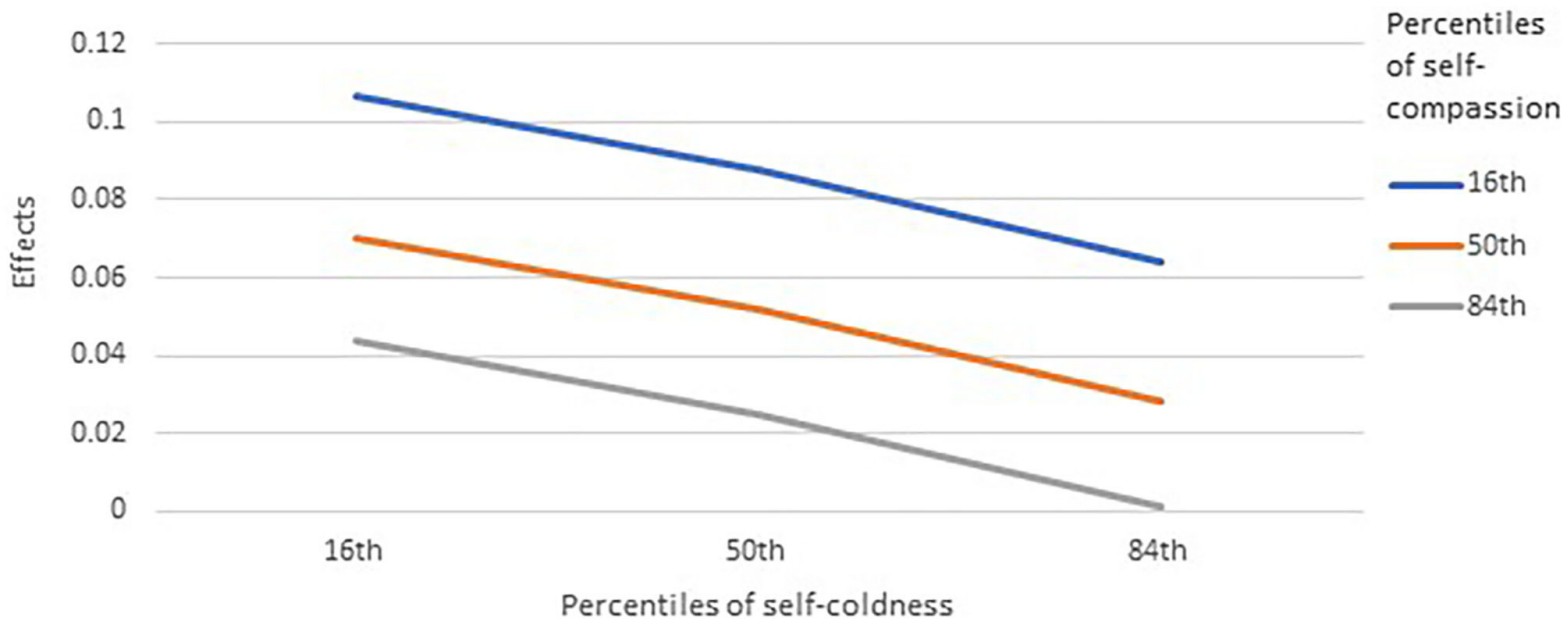
Moderation roles of self-compassion and self-coldness on the effects between perceived threats and perceived benefits (*N* = 761). Effects were unstandardized coefficients of the conditional effects of perceived threats on perceived benefits.

For perceived benefits, the main effect of self-compassion was significant, meaning that higher self-compassion was related to more perceived benefits, whereas that from self-coldness was non-significant. The main effect of perceived threat remained significant but positive, indicating more perceived benefits from higher levels of perceived threat. Only the moderation effect from self-compassion was significant (*p* = 0.0151). Inspecting the direction of the moderation, higher self-compassion was related to a weaker positive relationship between perceived threats and perceived benefits (Figure 2). In other words, in people with lower self-compassion, greater perceived threats instill more perceived benefits. However, in people with higher self-compassion, perceived benefits were weakly related to perceived threats. Higher self-coldness appeared to be related to weaker association between threats and benefits, but the moderation was non-significant.

## Discussion

Based on a bifactor model that distinguishes self-compassion from its negative counterpart—self coldness, our findings underscore the moderating roles of both constructs on how pandemic-related threats may impact well-being (26). Specifically, as hypothesized, self-compassion buffered, while self-coldness amplified, the association between perceived threats and psychological distress. We also anticipated that self-compassion would reduce, while self-coldness would intensify, the negative impacts of perceived threats on perceived benefits. However, first, the moderating role of self-coldness was not supported in our findings. Second, rather than diminishing a negative relationship between perceived threats and perceived benefits, self-compassion dampened a *positive* relationship between perceived threats and perceived benefits.

The findings on the buffering role of self-compassion and the amplifying role of self-coldness on the threat-psychopathology link echo with the conceptualization of the former as a protective factor in Neff and colleagues as well as the model of self-relating of Brenner et al. that views the latter as a risk factor (6, 26). Under both models, self-compassion may palliate the adverse impact of pandemic-related threats by enabling people to be kinder to oneself, evaluate the global threat as a shared experience with others and being mindful to one's needs. On the other hand, self-coldness may exacerbate psychological distress through further isolating one's pain from the fact that everyone is going through similar pains under the pandemic, forcing one to take more criticism than one's fair share in this macro catastrophe and hindering one from putting their difficulties in perspective.

In contrary to our expectation, more benefits were actually perceived in people facing more pandemic-related threats. The levels of perceived threats experienced by our participants were in general moderate. Such a level of threats would be threatening enough to trigger a response, but not too severe to have “frozen” the participants from responding or incurred resource loss so severe that adaptive coping strategies became impossible. Hence, greater threat perceptions could have triggered vigilance as well as cognitive and behavioral adaptations, which in turn enabled the discovery of benefits (41, 42). Our findings further suggest that those who scored higher on self-compassion experienced a weaker threats-benefits contingency than their counterparts who scored lower on that scale. Studies have noted people with high self-compassion were more inclined to using positive reinterpretation as a coping strategy and attuned to the positive aspects of their life even at the pre-conscious level (10, 43–45). Hence, our participants with high self-compassion were likely to have found benefits, regardless of their levels of perceived threats.

The relationship of self-coldness with perceived benefits might be less straight forward then the one with psychological distress. In the Asian culture that rewards modesty and emphasizes group harmony, being critical to oneself is not necessarily bad (46–48). Self-effacement may motivate self-improvement (49). That is, if benefit-finding is a way to improve oneself, individuals who have a tendency to self-efface may comply to win over the situation. Surely, if such efforts were ingenuine, there could be emotional costs. The line between of self-criticism due to adherence to social norms vs. self-disparagement is however often unclear (24).

### Study Limitations

Due to the unprecedented nature of COVID-19 pandemic, we relied on self-constructed measures to assess the degree of perceived threats imposed onto the participants' daily lives and the extent to which benefits and gains are experienced from the disrupted livelihood. As noted by Horesh and Brown, COVID-19 may represent a new type of mass trauma characterized by its global nature, lethality, novelty, and unpredictability, as well as the enormous anticipatory anxiety it ensues (50). As the pandemic appears to continue at least for a while with lasting aftermaths to our socio-economic-political landscape, psychologists should gather efforts to conceptualize the similarities and differences of the threats and benefits experienced by people under this global catastrophe, as compared to those of the victims of other disease outbreaks and disasters. Also, as the sample was non-random, the generalizability of our findings to the general population could be compromised. Specifically, males and individuals with lower socio-economic statuses were under-represented in this survey that took about 20–30 min to complete. COVID-19 could hit particularly hard on people without a financial and social safety net to fall on, including gig workers, individuals with physical or psychological disability and their caregivers, people living in poverty, and ethnic minorities. The suspension of support services and the worsening economic outlook means immense threats to their already-challenging lives. Thus, our estimates of psychological distress and perceived threats could be underestimates.

### Practical Implications

Mental health scientists are calling for studies on the causal and modifiable psychological factors that foster people's coping in the pandemic (51). Our findings point to the need to not only enhance the protective factors, such as self-compassion, but also to alleviate risk factors, such as self-coldness. Ferrari et al. conducted a meta-analysis with 27 randomized controlled trials and found that self-compassion interventions may result in a large effect size for rumination and moderate effect sizes for self-compassion, stress, depression, self-criticism, mindfulness, and anxiety, with sustained effects on self-compassion gains (52). Studies have also shown that regular but brief compassion meditation training via mobile applications and webpages can enhance well-being and self-compassion (53, 54). These interventions can be adapted to reach a larger audience during the pandemic using online channels. Based on the bifactor model of self-relating, therapists should explore means to, on one hand, facilitate a compassionate attitude to self, and on the other hand, alleviate toxic self-criticism, excessive rumination, and isolation (26).

## Conclusions

The COVID-19 pandemic is a humbling experience for many of us. Instead of a relentless chase after self-enhancement and self-esteem, acknowledging one's limitations as a part of the shared human experience with compassion could be particularly salutogenic, especially in such an unprecedented, challenging time. Our findings highlight the role of self-compassion to buffer the adverse consequences of perceived threats on well-being and to facilitate a general tendency to find benefits regardless of threats. Our findings also caution mental health professionals against the detrimental effects of self-coldness, as it may amplify psychological distress from perceived threats.

## Data Availability Statement

The raw data supporting the conclusion of this article will be available upon request to the corresponding author.

## Ethics Statement

The studies involving human participants were reviewed and approved by Human Research Ethics Committee of University of Hong Kong. The patients/participants provided their written informed consent to participate in this study.

## Author Contributions

BL conducted the data analysis. All authors contributed significantly to the conception, data collection, and writing up of the study.

## Conflict of Interest

The authors declare that the research was conducted in the absence of any commercial or financial relationships that could be construed as a potential conflict of interest.
